# Controlled deposition and combing of DNA across lithographically defined patterns on silicon

**DOI:** 10.3762/bjnano.4.8

**Published:** 2013-01-31

**Authors:** Zeinab Esmail Nazari, Leonid Gurevich

**Affiliations:** 1 Institute of Physics and Nanotechnology, Aalborg University, 9220 Aalborg, Denmark

**Keywords:** AFM, DNA molecular combing, DNA–peptide complexes, molecular electronics, surface modification

## Abstract

We have developed a new procedure for efficient combing of DNA on a silicon substrate, which allows reproducible deposition and alignment of DNA molecules across lithographically defined patterns. The technique involves surface modification of Si/SiO_2_ substrates with a hydrophobic silane by using gas-phase deposition. Thereafter, DNA molecules are aligned by dragging the droplet on the hydrophobic substrate with a pipette tip. Using this procedure, DNA molecules were stretched to an average value of 122% of their contour length. Furthermore, we demonstrated combing of ca. 900 nm long stretches of genomic DNA across nanofabricated electrodes, which was not possible by using other available combing methods. Similar results were also obtained for DNA–peptide conjugates. We suggest this method as a simple yet reliable technique for depositing and aligning DNA and DNA derivatives across nanofabricated patterns.

## Introduction

DNA is the subject of many investigations in different areas of nanotechnology research, ranging from genomic and biological studies [1–2] to the development of nanomachines and nanocircuits [3]. However, native double-stranded (ds) DNA is a flexible polymer that forms a random coil in aqueous solutions, hence hindering direct access for investigations and manipulations on DNA molecules unless they are straighten and immobilized on an appropriate substrate. In this context, Bensimon et al. introduced a so-called molecular combing technique in 1994 as an effective way to achieve ordered alignments of DNA molecules stretched on a solid surface [4]. The alignment occurs in two major steps: first, a random-coiled dsDNA floating in solution is partially melted at the ends. The ends with the exposed hydrophobic core are then readily adsorbed to the hydrophobic surface, hence anchoring the DNA molecules. Second, the meniscus is moved and the movement of the receding air–water interface leaves DNA behind, stretched on the dry substrate [4–6]. Once DNA is deposited and stretched on the surface, a wide variety of further manipulations on DNA become possible [7].

A number of different protocols have been devised based on the original technique proposed by Bensimon et al. They involve evaporation of a DNA solution [8–9], pulling a functionalized coverslip out of a DNA solution [10], using a filter paper [11], using a flow of nitrogen gas [12], pipette sucking [13], etc. Furthermore, several methods have been introduced that involved a combination of molecular combing with other techniques such as lithographic patterning [7]. For instance, Guan et al. used a combination of molecular combing with contact printing and soft lithography. With this method, it was possible to generate complex patterns of DNA on the substrate [14]. An important advantage of the combing method is that it does not require any prior modification of DNA. This makes it an excellent choice for the stretching of DNA on solid substrates for a variety of different applications. In addition to extensive applications in physics and nanoelectronics [7–8,15–16], many biomedical and genomic studies employ molecular combing as an effective tool for the generation of highly ordered alignments of DNA for various investigations, including gene mapping, DNA sequencing, and analysis [17–18].

**Figure 1:**
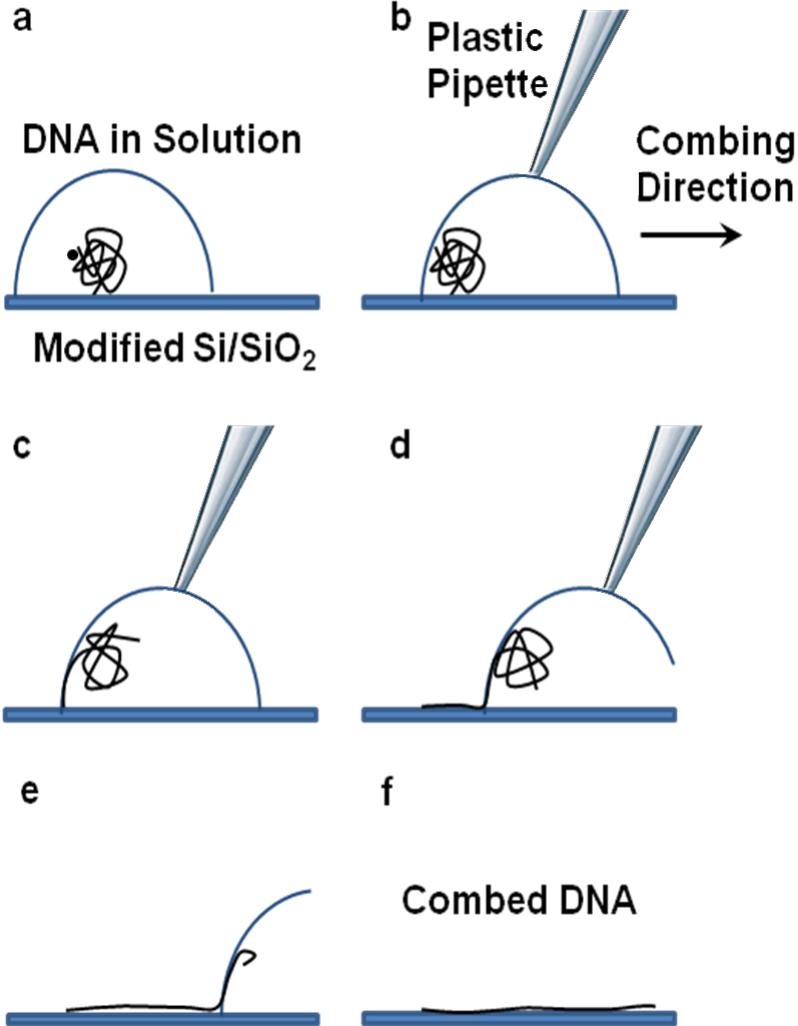
Schematic representation of the new combing method. The droplet containing the DNA solution makes contact angles of around 90° with the hydrophobic *N*-octyldimethylchlorosilane coated silicon surface. At pH 5.1 and ionic strength of 100 mM, DNA is adsorbed to the surface by the ends. By using a plastic pipette tip, the droplet is gently dragged out of the surface. The movement of the air–water interface results in the stretching of DNA molecules, which are fixed to the surface by one or both ends.

Most combing methods reported so far involve substrates such as mica, glass, plastic, etc., which are more convenient for DNA deposition and DNA studies, whereas only a few have attempted to adapt the technique to silicon surfaces [11]. However, since silicon is the most common material in micro- and nanofabrication, the dream of DNA-based chips [15] will not come true unless techniques for the manipulation of DNA are optimized for silicon substrates. This inspired us to develop a more “silicon-technology-friendly” variation of a combing method that involves the use of modified silicon substrates and lithographic methods. In this procedure, silicon substrates are coated with a thin layer of a hydrophobic silane by gas-phase deposition. Figure 1 is a representation of the combing procedure used in this experiment.

In the method proposed here, the applied meniscus force is large enough to allow efficient combing of DNA across nanofabricated patterns as well. Stretching of DNA across nano-electrodes has been previously achieved by methods such as electric field immobilization [19–20]; yet no report has been published on the immobilization of DNA on electrodes by molecular combing. We also successfully applied this combing technique to achieve stretching of various DNA–peptide conjugates. These nanomaterials have been recently prepared by our team and are composed of a dsDNA core and peripheral coating layer of self-assembled cationic peptides [21–22].

## Results and Discussion

**Figure 2:**
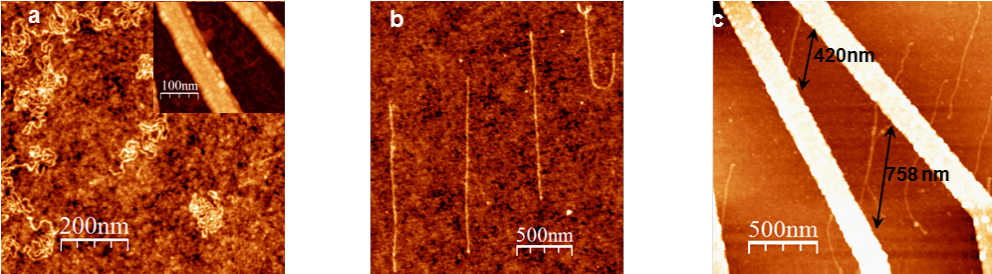
AFM topographic images of dsDNA molecules deposited on silicon substrates. (a) DNA deposited on APTMS-functionalized silicon substrates. The insert shows DNA in the area with nanoelectrodes. (b) DNA molecules combed on hydrophobically modified silicon substrates by using the proposed variation of the combing method. (c) DNA combed across nanofabricated electrodes. The typical observed dsDNA height was 0.7 ± 0.2 nm, in line with other experiments.

As was mentioned above, DNA molecules acquire a relatively compact coiled configuration in aqueous solution. If DNA is attracted from the solution towards the surface, e.g., electrostatically by introducing positive charge on the surface by APTMS functionalization, the final geometry of DNA molecules on the surface reflects this coiled configuration, as shown in Figure 2a. Deposition of DNA molecules across the electrodes is problematic in this case, even for relatively long DNA (the contour length of the DNA used was about 900 nm assuming B-DNA conformation). This situation is further aggravated by the fact that the negatively charged DNA is predominantly attracted to the positively charged modified area between the electrodes. On the other hand, as demonstrated in Figure 2b, the new variation of the combing method resulted in highly aligned DNA molecules oriented along the direction of the moving meniscus in an orderly and highly reproducible fashion. The average percentage of stretching was calculated as 122%, which is comparable to most values reported in earlier studies [23]. Using the new procedure, it was also possible to comb DNA across fabricated nanostructures, as shown in Figure 2c.

**Figure 3:**
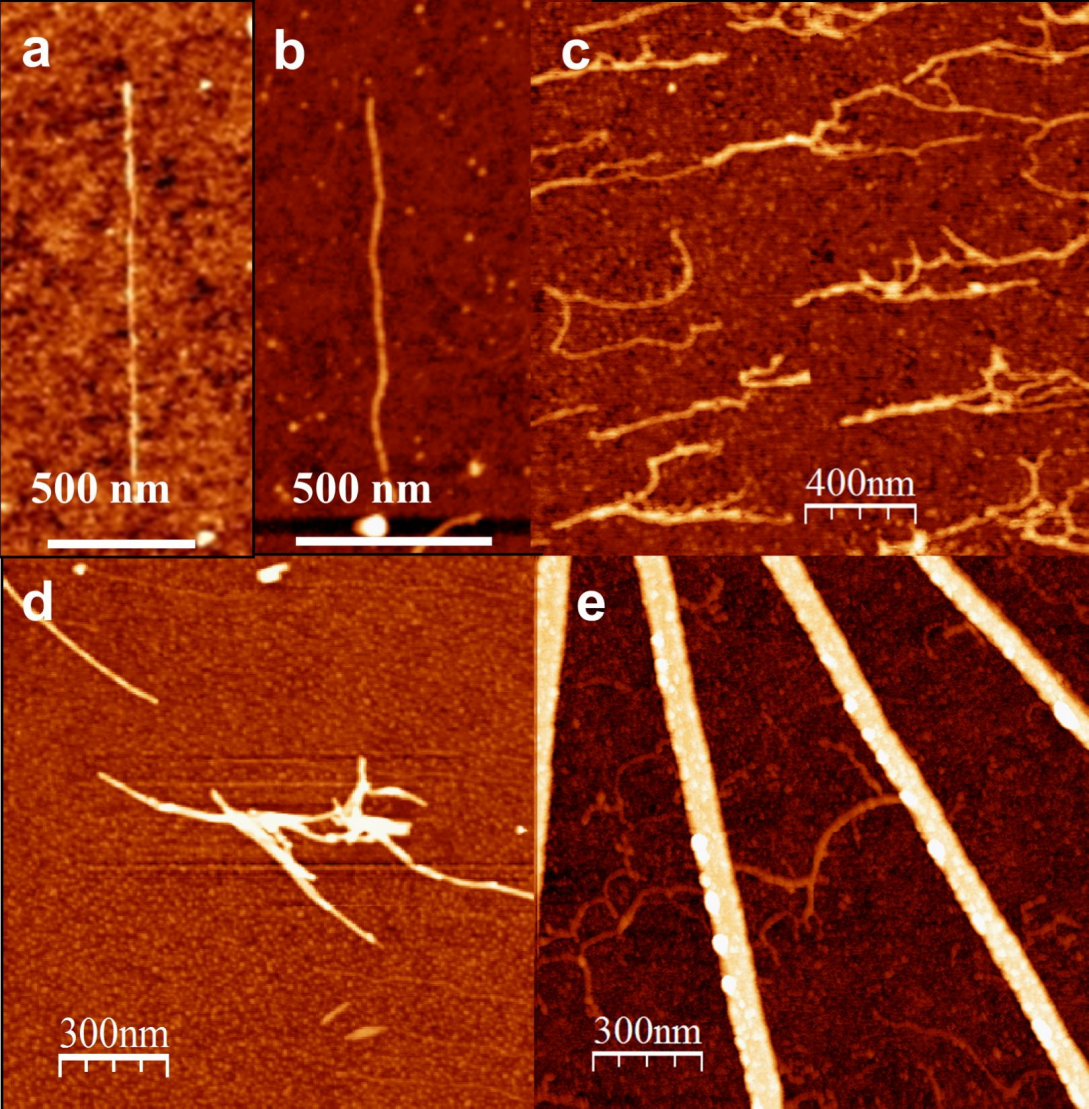
AFM topographic images demonstrating combing of dsDNA and DNA–peptide conjugates on hydrophobically modified silicon substrates. Vertical scale varies for different images. (a) Single dsDNA molecule, shown for comparison. (b) Single DNA–KA_6_ conjugate, height 3.4 ± 0.4 nm. (c) IL-coated DNA molecules aligned in the direction of combing; typical height is 2–5 nm depending on the bundle size. (d) DNA–KA_5_ conjugates; typical height of a single complex 5.6 ± 0.4 nm. (e) DNA–KA_6_ conjugates combed across nanoelectrodes; height 3–5 nm depending on the bundle size.

Interestingly, the new method was also efficient in combing DNA–peptide conjugates, while the original recipe was proven to be ineffective for combing these materials [11]. Figure 3 represents the topography of combed dsDNA conjugated with various peptides. Combing across nanoelectrodes was also possible for DNA–peptide conjugates (Figure 3e).

The gas-phase deposition of *N*-octyldimethylchlorosilane on silicon substrates used in this study was a key step to achieve hydrophobic and clean surfaces, ideal for deposition and combing of DNA. This procedure did not increase substrate roughness (average RMS ≈ 0.25 nm on modified substrates versus average RMS ≈ 0.3 nm before gas-phase deposition). Interestingly, on the nanoelectrodes, the observed density of deposited and combed DNA was significantly lower than that on flat silicon. This could be mainly attributed to the absence of silane functionalization on the platinum electrodes, which is also indicated by the absence of an additional tunnel barrier observed in [21–22].

The proposed variation of the combing method resulted in significant improvement in the quality of combed dsDNA on silicon. In addition to achieving more ordered alignments, we found the new method to be highly reproducible. We also observed that there is a narrow range of pH between 5.0 and 5.5 required for successful deposition and combing on the hydrophobic substrate, which was in agreement with earlier reports on combing [8–9]. The new procedure was also effective in combing DNA–peptide conjugates, while other commonly used combing recipes were ineffective for combing these materials. In the case of nanoelectrodes, despite the fact that the movement of the meniscus is disturbed when passing over the electrodes, DNA molecules were still combed across them in the desired direction, i.e., perpendicular to the electrodes.

## Conclusion

In this study, we combined gas-phase deposition and lithographic methods with a new variation of the combing technique in order to achieve high-quality alignments of DNA both on a flat silicon dioxide surface and across nanoelectrodes. The gas-phase deposition procedure together with choice of the buffer and *N*-octyldimethylchlorosilane to modify the surface provided optimal conditions for stretching of DNA up to 160% of its original contour length. The average percentage of stretching was calculated as 122%, which corresponds to the combing force of ≈2.4 nN. Furthermore, it was possible to achieve 900 nm long stretches of dsDNA deposited across nanoelectrodes. Not only successful in combing dsDNA with high quality and reproducibility, the new technique was also able to comb a number of DNA derivatives, which was not possible with other combing methods. The results of this study offer an efficient and reliable method for the aligned deposition of DNA and DNA derivatives for further applications in DNA nanotechnology.

## Experimental

The experiments were performed on a “random” sequence genomic DNA (pUC19/SmaI digest, 25 ng/µL, Fermentas Life Sciences). Pure ammonium acetate solution (20 mM, pH 5.1, Sigma Aldrich) was used as a buffer in all the procedures described here. The DNA solution was buffer exchanged to ammonium acetate before use, to guarantee that only “volatile” ions are present on the substrate. The following peptides were used to form DNA–peptide conjugates: indolicidin, abbreviated as IL, (Ile-Leu-Pro-Trp-Lys-Trp-Pro-Trp-Trp-Pro-Trp-Arg-Arg), IL4 (Ile-Leu-Pro-Trp-Lys-Leu-Pro-Leu-Leu-Pro-Leu-Arg-Arg), KA_5_ (Lys-Ala-Ala-Ala-Ala-Ala) KA_6_ (Lys-Ala-Ala-Ala-Ala-Ala-Ala), and KA_6_W (Lys-Ala-Ala-Ala-Ala-Ala-Ala-Trp). All the peptides used in the experiments were produced in-house by using solid-phase synthesis (Activo-P11, Activotec) and purified by HPLC before usage.

Two types of substrates were used during this experiment: clean silicon substrates (highly doped p-type silicon with 100 nm of thermal oxide, Nova Wafers, USA), as well as those with nanofabricated electrodes. Nanoelectrodes were fabricated by using a combination of optical and e-beam lithography followed by lift-off. In this way, we could achieve thin (5–10 nm) continuous Pt/Cr electrodes with a width of 30–40 nm and electrode spacing down to 40 nm. Prior to functionalization, both types of substrates were thoroughly cleaned and treated for 15–20 min in UV-ozone cleaner (BioForce Nanoscience). For gas-phase deposition, a solution of *N*-octyldimethylchlorosilane (Sigma-Aldrich) in toluene (1:3) was introduced into an evacuated chamber (≈100 mbar) containing the substrates for two hours. This resulted in a thin film of the hydrophobic silane on top of the SiO_2_ layer. On these surfaces, water droplets exhibited average contact angles of about 90° as determined by the sessile droplet method. Positively charged silicon surfaces (used for the reference experiment shown in Figure 2a) were produced by the same technique but with 3-aminopropyltrimethoxysilane (Sigma-Aldrich) and one hour incubation time.

The optimal conditions for combing were achieved with 20 mM ammonium acetate at pH 5.1 and *N*-octyldimethylchlorosilane surface modification. Molecular combing of DNA was performed according to the following procedure: A droplet of dsDNA solution in buffer (with the final concentration corresponding to absorption at 260 nm wavelength, *A*(260 nm), in the range 0.001–0.01, depending on the density of molecules on the surface required) was deposited on a silanized substrate followed by ≈6 min incubation time at room temperature. On a sufficiently functionalized substrate, the droplet produces a contact angle of ≈90°, which makes it easy to gently move the droplet along the surface. In this experiment, we used a plastic pipette tip to drag the droplet out of the substrate.

Preparation of DNA–peptide conjugates was performed in two different ways depending on the peptide. For peptides KA_5_, KA_6_, and KA_6_W the following procedure was used: The stock peptide solutions of KA_5_ (8 mM), KA_6_ (4 mM), and KA_6_W (4 mM) in buffer were sonicated for 30 min prior to mixing in a ratio 2:1 with DNA solution (*A*(260 nm) ≈ 0.05), followed by 2 h incubation of the mixture at room temperature. Combing of the DNA–peptide solution was performed with the same method as described for dsDNA but with longer (8–10 min) incubation times.

Combing of DNA conjugates with IL and IL4 was performed in two steps. First, DNA solution (*A*(260 nm) ≈ 0.05) was combed on a silanized substrate as described before. Then, the substrates were treated again with a second droplet containing peptide solution (8 min incubation). The droplet was then dragged out of the surface by the same combing technique, in the same direction. IL (680 µM) and IL4 (20 µM) were used without sonication. The same procedure was carried out in order to deposit and comb DNA and DNA–peptide conjugates on platinum nanoelectrodes.

Atomic Force Microscopy (AFM) was carried out on a Nanoscope IIIa (Bruker, USA), operating in tapping mode. OMCL-AC200TS, OMCL-AC240TS (Olympus), and HR-SCC (Team Nanotec GmbH) cantilevers were used for AFM imaging. The images were processed by using the WSxM software package [24].
